# Intra-arterial EmboCept S® and DC Bead® effectively inhibit tumor growth of colorectal rat liver metastases

**DOI:** 10.1186/s12885-019-6135-x

**Published:** 2019-10-10

**Authors:** Christian Ziemann, Jonas Roller, Markus M. Malter, Kira Keller, Otto Kollmar, Matthias Glanemann, Michael D. Menger, Jens Sperling

**Affiliations:** 1 Institute for Clinical and Experimental Surgery, Saarland University, Homburg/Saar, Germany; 2 Department of General, Visceral, Vascular and Pediatric Surgery, Saarland University, Homburg/Saar, Germany; 3 Present address: Department of Cardiovascular Surgery, University Heart Center, University Medical Center, University of Freiburg, Hugstetter Str. 55, 79104 Freiburg, Germany; 4 Present address: Department of General and Visceral Surgery, Helios Dr. Horst Schmidt Kliniken, Wiesbaden, Germany; 5 Present address: Department of General and Visceral Surgery, University Medical Center Göttingen, Göttingen, Germany

**Keywords:** Intra-arterial therapy, Degradable starch microspheres, Colorectal liver metastasis, Necrosis, Angiogenesis

## Abstract

**Background:**

Intra-arterial therapy with embolics is established for the treatment of malignancies of the liver. However, there are no studies comparing the different effects of various embolics used in clinical practice. Herein, we analyzed the effect of 3 different embolics on tumor growth in a rat model of colorectal liver metastases.

**Methods:**

Eight days after subcapsular implantation of 5 × 10^5^ colorectal cancer cells (CC531) in the left liver lobe of WAG/Rij rats were randomized into 4 groups (*n* = 8) and underwent intra-arterial hepatic therapy. Animals received either EmboCept S®, DC Bead® or Lipiodol® Ultra-Fluid. Animals of the control group received a comparable amount of saline. Tumor growth was measured on day 8 and 11 using a three-dimensional 40 MHz ultrasound device. On day 11 tumor and liver tissue were removed for histological and immunohistochemical analyses.

**Results:**

On day 11 animals of the control group showed a tumor growth of ~ 60% compared to day 8. Application of Lipiodol Ultra-Fluid® did not significantly influence tumor growth (~ 40%). In contrast, treatment with EmboCept S® or DC Bead® completely inhibited tumor growth. Of interest, application of EmboCept S® did not only completely inhibit tumor growth but even decreased tumor size. Immunohistochemical analysis showed a significant increase of necrotic areas within the tumors after application of EmboCept S® and DC Bead® compared to Lipiodol® Ultra-Fluid.

**Conclusion:**

The present study demonstrates that an intra-arterial therapy with EmboCept S® and DC Bead®, but not Lipiodol® Ultra-Fluid, results in a complete inhibition of rat colorectal liver metastatic growth.

## Background

Colorectal cancer (CRC) is the second most leading cause of cancer death worldwide. The liver is the most common site of distant metastases [1]. The treatment of colorectal liver metastases is challenging, because only 15–20% of patients are eligible for resection [2]. Surgical resection is the only treatment modality that potentially provides cure. In patients with unresectable metastases, systemic chemotherapy (SCT) remains the standard therapy. SCT has the potential to downsize the tumor volume so that surgical resection might become feasible. Unfortunately, SCT cannot achieve cure in these patients and is limited by systemic toxicity. Currently, a combination of 5-FU, folonic acid and either irinotecan (FOLFIRI) or oxaliplatin (FOLFOX) is the standard first-line therapy of metastatic CRC. However, the response rate is only 49% and there is a high rate of progression during the first 7 months [3]. The combination of FOLFIRI or FOLFOX therapy with bevacizumab improved the response rate up to 60% with a progression free survival of 10–12 months [4].

In case of unresectability and poor response to SCT, the median survival of patients suffering from CRC liver metastases is reported between 6.9 and 14.2 months [5]. In this context, intra-arterial therapies might represent an alternative strategy. Intra-arterial therapies for unresectable hepatic metastases from colorectal cancer include radioembolization (RE), transarterial chemoembolization (TACE) and hepatic arterial chemotherapy [6]. In other tumor entities like hepatocellular carcinoma (HCC), TACE already is an established therapeutic procedure [7]. TACE has the potential to control growth of HCC or even downsize the tumor volume. Colorectal liver metastases might also represent a target for intra-arterial therapies [6]. Currently, there is an ongoing discussion which chemoembolic has the highest efficiency and is more favourable for the patient [8]. However, there is no data on the effect of the different embolics. Therefore, we analyzed from the point of view of current challenges in CRC in an experimental animal study different clinical used embolics and their effectiveness. We herein compared in a rat model of colorectal liver metastases Lipiodol® Ultra-Fluid (LIP), EmboCept S® (EMB) and DC Bead® (DCB) for their effectiveness on tumor growth and for their side effects [9].

## Methods

### Animals

For the experiments, we used 32 WAG/Rij rats with a body weight of 184 ± 12 g. Animals were randomized in the following four groups (*n* = 8 each): 1. Control group (Sham), 2. Lipiodol® Ultra-Fluid (LIP), 3. EmboCept S® (EMB) and 4. DC Bead® (DCB). The animals were harbored in a temperature- and humidity-controlled 12 h light/dark cycle environment with free access to water and standard laboratory chow. All experiments were authorized by the local governmental ethic committee and were performed in accordance to the UKCCCR guidelines for the welfare of animals in experimental neoplasia [10] and the Interdisciplinary Principles and Guidelines for the use of animals in research. All animals were provided by Charles Rivers, Sulzbach, Germany. At the end of the experiments the animals were euthanasied by an overdosage of phenobarbital.

### Tumor cell implantation

For establishment of liver metastases, the animals were positioned in dorsal recumbency on an electronically regulated heating pad, which kept the body temperature at 37 °C. After median laparotomy under isoflurane anesthesia, 5 × 10^5^ cells of syngeneic CC531 colon carcinoma cells were implanted under the capsule of the left liver lobe using a 27G needle (Omnicon F, Braun, Melsungen, Germany). The laparotomy was closed by a two-layer running suture (Prolene 3–0, Ethicon/Johnson & Johnson MEDICAL GmbH, Norderstedt, Germany).

### Catheter placement for intra-arterial therapy

Eight days after tumor cell implantation, a relaparotomy was performed. The gastroduodenal side branch of the common hepatic artery was cannulized with a catheter (ID 0.28 mm, Portex, Hythe, UK). During the treatment the common hepatic artery was not obturated and orthograde blood flow was observed. After the treatment the catheter was unplaced and the gastroduodenal artery was ligated.

### Intra-arterial therapy

EMB (PharmaCept GmbH, Berlin, Germany) contains degradable starch microspheres with a median size of 50 μm. EMB has a half-life period of 35 min. EMB was given in a dose of 6.43 mg/kg body weight. DCB (Terumo Deutschland GmbH, Eschborn, Germany) are non-degradable polyvinyl alcohol hydrogels. DCB with a size of 70-150 μm was administered in a dose of 0.14 ml/kg. LIP (Guerbet LLC, Bloomington, USA) is an iodized oil (median size 2-3 μm) made from poppy-seed oil (oleum papaveris) and was given in a dose of 0.15 ml/kg body weight. LIP has a half-life period of about 7 days. Animals of the control group received a comparable amount of saline solution (Sham).

### Three-dimensional ultrasound imaging

The 40 MHz ultrasound probe of the Vevo 770 high-resolution imaging system (Visual-Sonics, Inc., Toronto, Ontario, Canada) was utilized for ultrasound imaging. Ultrasound imaging was achieved in situ directly on the liver surface on day 8 and 11. During imaging, animals were anesthetized with isoflurane and strapped on a heated stage. To achieve three-dimensional imaging, parallel two dimensional images were obtained in 50 μm intervals controlled by a stepping motor. For three-dimensional reconstruction of the tumor the data was outlined in two-dimensional images (200 μm) offline. These data was utilized by the integrated software of the ultrasound device to achieve a three-dimensional image and calculate the tumor volume.

### Sampling and assays

On day 8 and 11 before ultrasound imaging venous blood samples were acquired via puncture of the subhepatic vena cava. In addition, white blood cell count, hemoglobin and platelets were studied.

### Histology

Tissue specimens of the tumor bearing livers were fixed in 4% phosphate-buffered formalin for 2–3 days at the end of each experiment. These Specimens were imbedded in paraffin. For histological analysis of hepatocellular injury, venular endothelial detachment and venular fibrin clotting sections (5 μm) were cut and stained with hematoxylin-eosin. Hepatocellular injury was defined by analysis of hepatocellular vacuolization with a semiquantitative score, i.e. (0: none; 1: mild; 2: moderate; 3: severe). Venular endothelial detachment was evaluated by counting the number of venules with detachment of endothelial lining cells per HPF. To indicate venular fibrin clotting, the number of venules with fibrin clots were given in percent of all venules. Necrotic areas in the tumor tissue were measured as percentage of the whole tumor area. For analysis of the inflammatory response leukocyte accumulation was determined in normal liver tissue using chloroacetate esterase staining. The inflammatory response was assessed by counting the number of leukocytes per HPF.

### Immunohistochemistry

PCNA (proliferating cell nuclear antigen) were used as an indicator of cell proliferation. Paraffin-embedded specimens of 5 μm were incubated for 18 h at 4 °C with a rabbit polyclonal anti-PCNA antibody (1:50; Santa Cruz Biotechnology Inc., Heidelberg, Germany). For development of PCNA, an alkaline phosphatase-conjugated goat anti-rabbit IgG (1:20; Dako Cytomation, Glostrup, Denmark) was incubated for 30 min. Fuchsin (PCNA) served as chromogen and hemalaun served for counterstaining. An index ranging from 0 to 4 of PCNA-positive cells (0: < 1%, 1: 1–10%, 2: 10–30%, 3: 30–50%, 4: > 50% of PCNA-positive cells) were used to analyze these sections.

Cleaved caspase-3 (cysteine-aspartic proteases) was used as an indicator of apoptotic cell death. Five μm sections of paraffin-embedded tumor-bearing liver specimens were incubated overnight at room temperature with a rabbit polyclonal anti-cleaved caspase-3 antibody (1:100, New England Biolabs, Frankfurt, Germany). This antibody identifies endogenous levels of the large fragment (17/19 kDa) of activated caspase-3, but not full length caspase-3. For streptavidine-biotin complex peroxidase staining, a biotinylated anti-rabbit Ig antibody served as secondary antibody (ready-to-use, Abcam, Cambrige, UK). 3.3′ diaminobenzidine served as chromogen. Sections were counterstained with hemalaun. In tumor and normal liver tissue positively stained cells were numbered in 25 HPF per specimen, and are indicated as number per HPF.

PECAM-1 (platelet-endothelial cell adhesion molecule-1; CD31) is expressed exclusively on endothelial cells and can function as an indicator for vascularization. For immunohistochemical identification of PECAM-1 expression a primary mouse-anti-rat antibody (1:250; BioRad, Puchheim; Germany) as well as a crossreacting, peroxidase-conjugated goat-anti-mouse antibody (1:200; BioRad, Puchheim, Germany) was used. PECAM-1-positive blood vessels were numbered in 25 HPF per section and are indicated as number per HPF.

### Statistical analysis

All values are given as mean ± SEM. After analysis of normal distribution of data and homogeneity of variance, differences between the groups were evaluated by an one-way analysis of variance (ANOVA) followed by an appropriate post hoc test, including the correction of the alpha-error according to Bonferroni probabilities. Overall statistical significance was set at *p* < 0.05. Statistical analysis was performed with the use of SigmaPlot 13.0. (Systat Software GmbH, Erkrath, Germany).

## Results

### Metastatic tumor growth and general health conditions

After relaparotomy at day 8 all animals showed an established tumor in the left liver lobe with a diameter of 5–10 mm. The pre-treatment tumor volumes showed consistent across treatment groups. There were no signs of peritoneal or other extrahepatic metastases. The malignant process did not affect animals systemically. In particular, they showed normal feeding and cleaning habits.

### Tumor growth

**Fig. 1:**
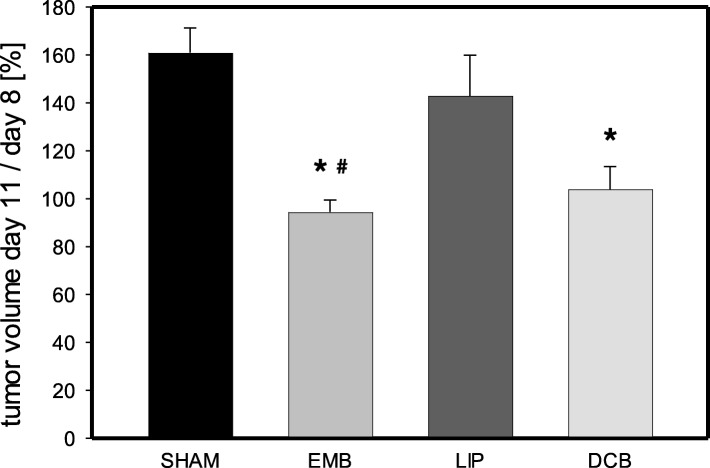
Tumor growth at day 11 (given as tumor volume in % of the tumor volume at day 8) in animals undergoing intra-arterial application of EmboCept S® (EMB), DC Bead® (DCB), Lipiodol® Ultra-Fluid (LIP) or saline solution (SHAM). Data are given as mean ± SEM; *p < 0.05 vs. SHAM; #*p* < 0.05 vs. LIP

Animals of the Sham group showed a 60% increase of tumor the volume from day 8 to day 11 (Fig. 1). Application of DCB and EMB completely inhibited tumor growth during this three-day period (Fig. 1). Of interest, EMB not only completely prevented tumor growth but additionally reduced the tumor size (Fig. 1). In contrast, LIP did not significantly affect tumor growth compared to Sham controls (Fig. 1).

### Tumor cell proliferation

Analysis of tumor cell proliferation did not show significant differences at day 11 between the groups in tumor tissue (Sham 3.2 ± 0.1; EMB 2.9 ± 0.1; LIP 3.0 ± 0.2; DCB 3.4 ± 0.2 (Score)) and normal liver tissue (Sham 0.6 ± 0.3; EMB 1.0 ± 0.4; LIP 0.6 ± 0.2; DCB 0.7 ± 0.3 (Score)).

### Apoptotic cell death

Analysis of apoptotic cell death at day 11 did not show significant differences between the groups in normal liver tissue (Sham 0.8 ± 0.1 per HPF; EMB 1.2 ± 0.2 per HPF; LIP 1.4 ± 0.2 per HPF; DCB 1.3 ± 0.2 per HPF).

### Necrotic cell death

**Fig. 2:**
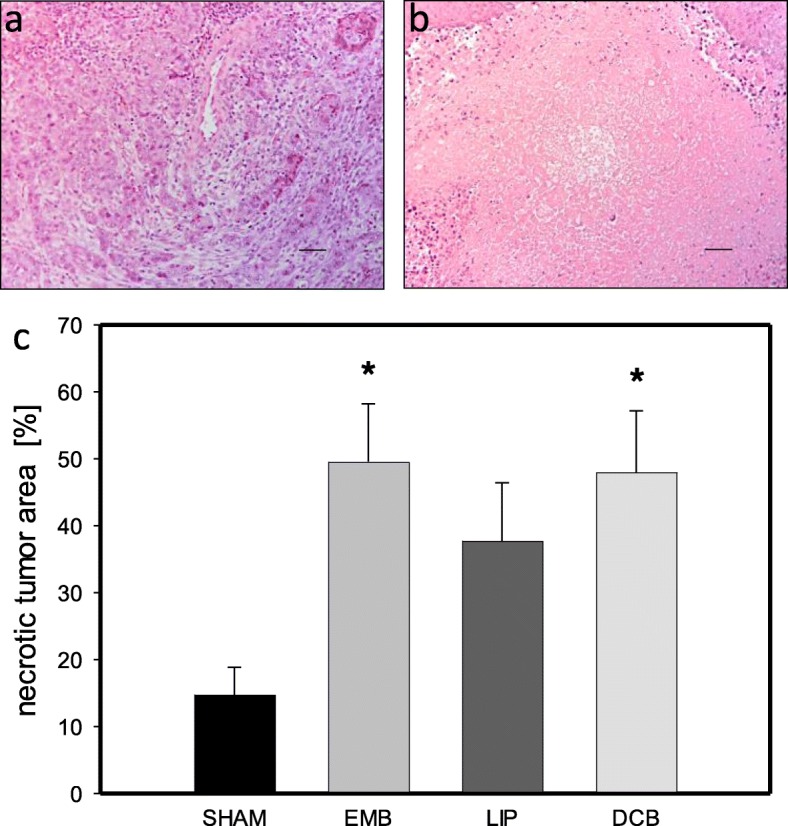
Histological analysis of necrotic cell death. **a** shows the tumor tissue of a SHAM animal. **b** displays tumor tissue of an EMB animal. **c** demonstrates the quantitative analysis of necrosis (given as necrotic area in % of total tumor area) in the tumor tissue of animals undergoing intra-arterial application of EmboCept S® (EMB), DC Bead® (DCB), Lipiodol® Ultra-Fluid (LIP) or saline solution (SHAM) on day 11. Data are given as mean ± SEM; *p < 0.05 vs. SHAM. Bars represent 100 μm

In the tumor tissue analysis of necrotic cell death revealed over 40% of necrotic area in DCB and EMB animals (Fig. 2). In contrast, LIP treatment did not induce a significant number of necrotic cells compared to Sham controls (Fig. 2).

### Tumor vascularization

**Fig. 3:**
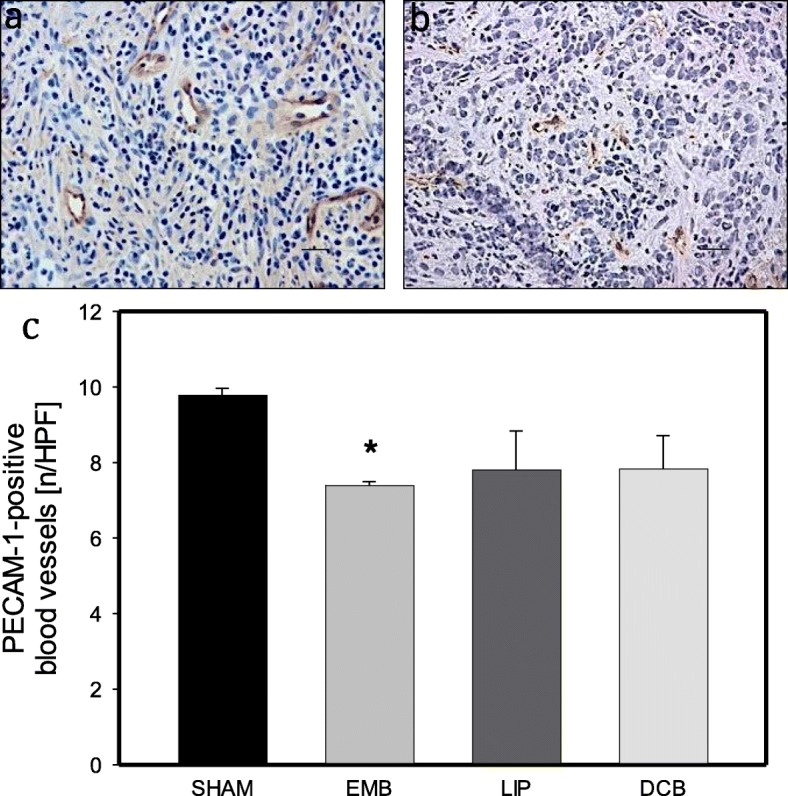
Immunohistochemical analysis of PECAM-1 as an indicator of vascularization of the tumor tissue. **a** shows a representative tumor section from a Sham control. **b** displays a tumor section of an EMB-treated animal. Note the lower number of PECAM-1-positive cells after EMB application compared to SHAM. **c** shows the quantitative analysis of PECAM-1-positive cells in tumors of animals undergoing intra-arterial application of EmboCept S® (EMB), DC Bead® (DCB), Lipiodol® Ultra-Fluid (LIP) or saline solution (SHAM). Data are given as mean ± SEM; *p < 0.05 vs. SHAM. Bars represent 50 μm

Analysis of tumor vascularization on day 11 revealed a slight decrease of PECAM-1-positive cells in tumors of EMB animals compared to Sham controls (Fig. 3). Of interest, LIP and DCB treatment were not capable of significantly reducing PECAM-1-positive cells compared to Sham controls (Fig. 3).

### Side effects

**Fig. 4:**
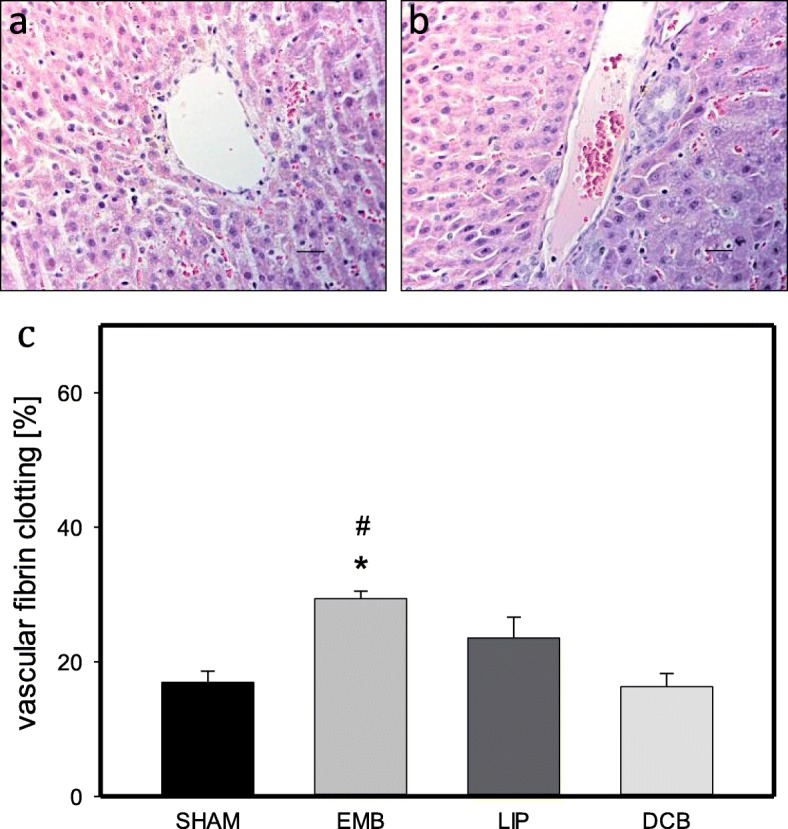
Histological analysis of venular fibrin clotting. **a** shows normal liver tissue of a Sham animal. **b** displays normal liver tissue of an EMB animal. **c** demonstrates the quantitative analysis of vessels with vascular fibrin clotting (given as vessels with fibrin clots in % of all vessels per HPF) in normal liver tissue of animals undergoing intra-arterial application of EmboCept S® (EMB), DC Bead® (DCB), Lipiodol® Ultra-Fluid (LIP) or saline solution (SHAM) on day 11. Data are given as mean ± SEM; *p < 0.05 vs. SHAM. Bars represent 50 μm

The animals did not show significant differences in body weight at day 11 given as percentage from that of day 0 (Sham 0.97% ± 0.27; EMB 0.94% ± 0.02; LIP 0.94% ± 0.02; DCB 0.93% ± 0.01). Histomorphological analysis of hepatocellular vacuolization could not demonstrate significant differences between the groups (Sham 1.24 ± 0.10; EMB 1.43 ± 0.23; LIP 0.97 ± 0.03; DCB 1.11 ± 0.14 (Score)). Vascular fibrin clotting revealed a significant increase after EMB treatment compared to Sham- or DCB-treated animals (Fig. 4). Venular endothelial detachment demonstrated a significant increase in EMB-treated animals compared to Sham- and DCB-treated animals (Sham 12.32% ± 0.61; EMB 31.36% ± 4.57; LIP 17.63% ± 2.96; DCB 13.25% ± 3.59).

Histomorphological analysis of the inflammatory response in the liver tissue using chloroacetate esterase staining was negligible after EMB or LIP treatment. Only DCB-treated animals showed distinct intravasal leukocyte accumulation in portal tracts (Table 1).

**Table 1:** Histological analysis of inflammatory response in normal liver

	sinusoids	portal tracts		venules	
		intravasal	extravasal	intravasal	extravasal
SHAM	0.25 ± 0.16	0.13 ± 0.13	0.38 ± 0.26	2.00 ± 0.73	1.88 ± 0.48
EMB	0.75 ± 0.31	0.75 ± 0.37	1.63 ± 0.91	2.00 ± 1.10	1.13 ± 0.44
LIP	1.13 ± 0.35	0.38 ± 0.18	1.25 ± 0.53	3.13 ± 0.55	1.88 ± 0.30
DCB	0.50 ± 0.27	1.50 ± 0.33 *	0.50 ± 0.19	3.88 ± 0.77	2.63 ± 0.63

Note 1.-Histological analysis of leukocyte accumulation in normal liver tissue given as leukocytes per HPF of animals undergoing intra-arterial application of EmboCept S® (EMB), DC Bead® (DCB), Lipiodol® Ultra-Fluid (LIP) or saline solution (SHAM) on day 11. Data are given as mean ± SEM; *p < 0.05 vs. SHAM

## Discussion

The major finding of the present study is that intra-arterial application of Embocept S® and DC Bead® is effective in reducing tumor growth of colorectal liver metastasis. This is most probably caused by increased ischemic tumor necrosis. Of interest, EMB does not only inhibit tumor growth, but additionally reduces tumor volume and decreases tumor vascularization.

It is well known, that colorectal liver metastases receive their nutritive blood supply predominantly from the hepatic arterial axis, whereas the liver parenchyma receives both portal venous and hepatic arterial blood [11–13]. Accordingly, we did not see an increased rate of apoptotic cells within the normal liver tissue after administration of embolics. This suggests, that the embolic particles do not compromise substantially nutritive blood flow of the normal liver tissue.

Administration of EMB and DCB caused a significant increase of the area of necrosis within the tumor tissue. In contrast, LIP did not show any increase of the necrotic area within the tumor tissue. This effect is most probably caused by the different size of the embolics used. The particle sizes of the embolics used in the present study are 35-70 μm (EMB), 70-150 μm (DCB) and 2-3 μm (LIP). While the diameters of the hepatic sinusoids range between 10 and 15 μm [14], capillaries of the hepatic arterial system exhibit mean diameters of 8–10 μm [15]. This indicates that a significant tumor embolization via the hepatic arterial system can only be achieved by particles with a size greater than the diameter of the hepatic capillaries. In line with the induction of necrotic cell death within the tumor tissue, we found that administration of EMB and DCB, but not LIP, significantly inhibited tumor growth at day 11. EMB may occlude terminal hepatic arterioles peripherally of shunt branching. This may guarantee an almost complete cessation of blood flow in the tumor, which is due to the arterial origin of its blood supply. The normal liver tissue preserves tissue oxygenation by both blood supply from the portal-venous axis and by shunting of oxygenated blood from the hepatic arterial axis. DCB consists of bigger particles that occlude the terminal hepatic arterioles centrally of shunt branching. This may maintain a reduced blood flow by portal-arterial shunting, resulting in a reduced tissue oxygenation because the blood supply originates solely from the less oxygenated portal-venous axis [11].

Kohli et al. have indicated about 50% apoptotic hepatocytes in rat livers after 60 min of warm ischemia and 24 h of reperfusion [16]. In contrast, Gujral et al. showed that cell death during warm hepatic ischemia and reperfusion is mainly due to necrosis [17]. In line with this, others confirmed that after 180 min of warm ischemia and 24 h of reperfusion cell death is predominantly induced by necrosis [11, 18]. This is in accordance with the results of the present study, demonstrating that tumor cell death is mainly induced by necrosis.

In addition to the tumor reduction by necrotic cell death, the size of the tumors in our study might have been influenced by effects of the embolics on tumor angiogenesis and tumor cell proliferation. In fact, recent studies showed in a hepatoma model in rats a reduced angiogenesis and proliferation after application of an embolic [19, 20]. Of interest, we could not detect any changes in the number of PCNA-positive cells, indicating that treatment with the different embolics in the present study did not influence tumor cell proliferation. However, we found that embolization with the degradable embolic particles (EMB) inhibited tumor angiogenesis, as indicated by a reduced number of PECAM-1-positive cells. Furthermore, EMB-treated animals showed a markedly increased injury of the endothelium of the postsinusoidal venules. It is well known, that the microvascular perfusion within the liver involves arterio-portal venular shunts to maintain homogeneity of nutritional supply. As shown before, the size of the arterioles forming shunts exert a diameter of about 50-100 μm [15]. In line with this, increased fibrin thrombi in postsinusoidal venules of EMB-treated animals might have been caused by the embolic, as EMB particles show a diameter below the size of the shunt arterioles. In contrast DCB are bigger than the shunt-building arterioles and unlikely to pass arterio-portal venular shunts.

## Conclusion

In conclusion, we demonstrate that intra-arterial embolization with EMB and DCB is effective to reduce tumor growth of colorectal liver metastasis. Accordingly, embolization with EMB and DCB may represent an effective intra-arterial therapy in unresectable colorectal liver metastasis. It is important to note that the present study is only an experimental animal study to evaluate current embolic particles without influence of any chemotherapeutics. Therefore, the current study has limitations in transfering its knowledge direct to clinical practice.

## Data Availability

The data analyzed during this study are included in this published article.

## Abbreviations

ANOVA: Assessed by a one-way analysis of variance
CRC: Colorectal cancer
DCB: DC Bead®
EMB: EmboCept S®
FOLFIRI: 5-FU, folonic acid and irinotecan
FOLFOX: 5-FU, folonic acid and oxaliplatin
HCC: Hepatocellular carcinoma
LIP: Lipiodol® Ultra-Fluid
PCNA: Proliferating cell nuclear antigen
PECAM-1: Platelet-endothelial cell adhesion molecule-1; CD31
RE: Radioembolization
SCT: Systemic chemotherapy
TACE: Transarterial chemoembolization
